# Receptor Interacting Protein 3-Mediated Necroptosis Promotes Lipopolysaccharide-Induced Inflammation and Acute Respiratory Distress Syndrome in Mice

**DOI:** 10.1371/journal.pone.0155723

**Published:** 2016-05-19

**Authors:** Linlin Wang, Tingting Wang, Haobo Li, Qing Liu, Zhongjun Zhang, Wanli Xie, Yinglu Feng, Tumenjavkhlan Socorburam, Gui Wu, Zhengyuan Xia, Qingping Wu

**Affiliations:** 1 Department of Anesthesiology, Union Hospital, Tongji Medical College, Huazhong University of Science and Technology, Wuhan, 430022, China; 2 Department of Anesthesiology, the Anesthesiology Research Center of Shenzhen city, Shenzhen People’s Hospital, Second Medical College of Jinan University, Shenzhen, 518020, China; 3 Department of Anesthesiology, The University of Hong Kong, Hong Kong SAR, China; 4 Emergency Department, Wuhan General hospital of Guangzhou Military, Wuhan, Hubei, China; University of Alabama at Birmingham, UNITED STATES

## Abstract

Necrosis amplifies inflammation and plays important roles in acute respiratory distress syndrome (ARDS). Necroptosis is a newly identified programmed necrosis that is mediated by receptor interacting protein 3 (RIP3). However, the potential involvement and impact of necroptosis in lipopolysaccharide (LPS)-induced ARDS remains unknown. We therefore explored the role and mechanism of RIP3-mediated necroptosis in LPS-induced ARDS. Mice were instilled with increasing doses of LPS intratracheally to induce different degrees of ARDS. Lung tissues were harvested for histological and TUNEL staining and western blot for RIP3, p-RIP3, X-linked inhibitor of apoptosis protein (XIAP), mixed lineage kinase domain-like protein (MLKL), total and cleaved caspases-3/8. Then, wild-type and RIP3 knock-out mice were induced ARDS with 30 mg/kg LPS. Pulmonary cellular necrosis was labeled by the propidium Iodide (PI) staining. Levels of TNF-a, Interleukin (IL)-1β, IL-6, IL-1α, IL-10 and HMGB1, tissue myeloperoxidase (MPO) activity, neutrophil counts and total protein concentration were measured. Results showed that in high dose LPS (30mg/kg and 40mg/kg) -induced severe ARDS, RIP3 protein was increased significantly, accompanied by increases of p-RIP3 and MLKL, while in low dose LPS (10mg/kg and 20mg/kg) -induced mild ARDS, apoptosis was remarkably increased. In LPS-induced severe ARDS, RIP3 knock-out alleviated the hypothermia symptom, increased survival rate and ameliorated the lung tissue injury RIP3 depletion also attenuated LPS-induced increase in IL-1α/β, IL-6 and HMGB1 release, decreased tissue MPO activity, and reduced neutrophil influx and total protein concentration in BALF in severe ARDS. Further, RIP3 depletion reduced the necrotic cells in the lung and decreased the expression of MLKL, but had no impact on cleaved caspase-3 in LPS-induced ARDS. It is concluded that RIP3-mediated necroptosis is a major mechanism of enhanced inflammation and lung tissue injury in high dose LPS- induced severe ARDS in mice.

## Introduction

Acute respiratory distress syndrome (ARDS) is a devastating clinical syndrome with high mortality (about 30%-50%)[1]. It is an inflammatory lung condition associated with the lung epithelium/endothelium injury and the malfunction of other organs due to the inability to take up oxygen[2]. Nowadays, many approaches have been proposed for the prevention and management of ARDS, however, the results have been disappointing. Different types of cell death including apoptosis, autophagy and necrosis, coexist to contribute to the development of ARDS[3]. Apoptosis has long been recognized as the only form of cell death that can be regulated and as such apoptosis has been extensively studied in development and progression of ARDS. Indeed, apoptosis has been reported as a predominant cell death pathway in ARDS and inhibition of apoptosis attenuates ARDS[4]. Unlike apoptosis and autophagy, which wrap intracellular contents within cells, necrosis features the release of cell lysis into the extracellular environment, and as a result, damage-associated molecular patterns (DAMPs) are released[5]. DAMPs, such as high-mobility group box 1 (HMGB1) and uric acid, can trigger or sensitize pattern recognition receptors (PRRs) to provoke inflammation[6]. Because inflammatory response plays an important role in ARDS, necrosis may accelerate tissue damage by promoting inflammatory response. However, the intervention of necrosis has largely not been studied in ARDS because previous studies reported that necrosis was uncontrollable and could not be specifically regulated.

Necroptosis is a newly identified type of cell death, which has the combination features of necrosis and apoptosis that can also be regulated. Recent studies suggest that necroptosis is mediated by receptor interacting protein 3 (RIP3)[7, 8]. Depletion of RIP3 abolished necroptosis. The execution of necroptosis needs RIP3 phosphorylation, while mixed lineage kinase domain-like (MLKL) was a substrate protein of RIP3[9, 10]. Studies have shown that X-linked inhibitor of apoptosis (XIAP), the prototype member in the inhibitor of apoptosis (IAP) family[11], could inhibit RIP3-dependent necroptosis and IL-1 activation[12]. However, the regulation of RIP3-dependent necroptosis in ARDS still remains unknown.

Necroptosis has been shown to be increased in several disease models, such as myocardial ischemia-reperfusion injury, cerulein-induced acute pancreatitis, renal ischemia-reperfusion injury, skin and intestinal inflammation and TNF-induced SIRS[13–15]. However, studies of necroptosis in the pulmonary system are rare. A recent study reported that toxin-induced necroptosis is a major cause of lung injury in staphylococcus aureus induced pneumonia[16]. Another study reported that necroptosis plays a role in influenza pneumonia only when the cellular inhibitor of apoptosis proteins (cIAP2) is absent[17]. The expression of RIP3 is increased in pulmonary endothelium in LPS-induced systematic vascular inflammation[18]. Red blood cell transfusion enhances the susceptibility to lung inflammation through the release of HMGB1 and the induction of necroptosis in lung endothelial cells[19].

Staphylococcus aureus is a kind of Gram-positive bacteria, and may induce different immune and inflammatory responses from Gram-negative bacteria. Lipopolysaccharide (LPS) is an important immunogenic component of the outer membrane of Gram-negative bacteria. LPS has been commonly used as a tool to study the mechanisms of ARDS both in animals and in cultured cells[20, 21]. However, whether necroptosis exists and has effect on LPS-induced ARDS remains unknown. Therefore, the current study was designed to explore the role of RIP3-mediated necroptosis and its underlying mechanism in a mouse ARDS model by instilling LPS intratracheally.

## Materials and Methods

### Reagents and antibodies

LPS 055:B5 was purchased from Sigma (St. Louis, MO, USA). Mouse RIP3 antibody was from Abcam (ab56164). Mouse p-RIP3 antibody and MLKL antibody were obtained from Prof. Jiahuai Han’s Lab in Xiamen University, China[22]. The GAPDH antibody, XIAP, caspase-3, cleaved caspase-3, caspase-8, and cleaved caspase-8 antibody were purchased from Cell Signaling Technology (CST5174). Horseradish peroxidase-conjugated goat anti-rabbit antibody, bicinchoninic acid (BCA) protein assay kit and enhancer chemiluminescent (ECL) reagent were purchased from Pierce Biotechnology (Rockford, IL, USA). The protein extraction kit and TUNEL in situ apoptosis detection kit were from Key GEN Bio TECH (Nangjing, China). Propidium iodide (PI) was from Sigma (P4170).

### Animals

This study was performed with the approval of the Wuhan University Medical Ethical Committee, China. Male C57/B6-mice (8–10 weeks, weight 25–30 g) were obtained from Wuhan University Center for Animal Experiments (Permit Number: SCXK2008–0004). All of the mice received humane care according to the guidelines of the Local Institutes of Health guide for the care and use of laboratory animals. RIP3^-/-^ mice, which were produced by TALENs-mediated gene disruption, were kind gifts from Prof. Jiahuai Han in Xiamen University, China.

### Experimental protocols

Mouse ARDS was induced by intratracheal instillation of LPS through a human intravenous catheter (24G) after anesthesia with ketamine (30 mg/kg) intraperitoneally. LPS was dissolved in 40 μl of PBS according to the needed concentration. Thirty mice were divided into six groups: (a) sham, (b) control, (c) LPS 10 mg/kg, (d) LPS 20 mg/kg, (e) LPS 30 mg/kg, and (f) LPS 40 mg/kg. The sham group mice were given endotracheal intubation but received neither PBS nor LPS, while the control group mice were given 40 μl of PBS after endotracheal intubation. Then wild-type (RIP3-WT) and RIP3 knock-out (RIP3-KO) mice were divided into four groups: (a) control RIP3-KO, (b) LPS treated RIP3-KO (30 mg/kg), (c) control RIP3-WT, and (d) LPS treated RIP3-WT (30 mg/kg). After 12 or 24 hours (h), the mice were sacrificed with dislocation of cervical vertebrae. The lungs were removed and immediately stored at -80°C until assayed.

### Pathological evaluation of lung injury

The left lung was obtained at the indicated times. Tissues were fixed in 4% paraformaldehyde and subsequently embedded in paraffin. Then, 4-μm sections were stained with HE using a standard protocol and analyzed by light microscopy. The severity of the lung injury was quantified as previously reported[23]. Lung injury was scored by a blinded pathologist according to the following four items: alveolar congestion, hemorrhage, infiltration or aggregation of neutrophils in the airspace or vessel wall, and thickness of the alveolar wall/hyaline membrane formation. Each item was graded according to a five-point scale: 0 = minimal (little) damage, 1 = mild damage, 2 = moderate damage, 3 = severe damage, and 4 = maximal damage. Thus, minimum and maximum possible scores were 0 and 16, respectively. The total lung injury score was calculated by adding up the individual scores of each category.

### Western blotting

The protein samples were prepared according to the protein extraction kit introductions. The protein concentrations were assessed using the BCA protein assay kit (Piece Biotechnology, Rockford, USA). In total, 80 mg of lung tissue was homogenized and lysed in 800 μl of RIPA buffer containing a protease and phosphatase inhibitor cocktail. Samples were boiled at 99°C for 10 minutes (min) and stored at -20°C until being used for immunoblotting. Then, 8-μl samples were run on 8% or 12% SDS/PAGE and transferred to a PVDF membrane. The membranes were then blocked for 2 h with 5% fat-free milk in TBST (0.1% Tween-20 in TBS), followed by incubation with rabbit primary polyclonal antibodies, including anti-RIP3 (1:1000), anti-p-RIP3 (1:1000), anti-MLKL (1:1000), anti-MLKL (1:1000), anti-caspase-3(1:1000), anti-cleaved caspase-3(1:1000), anti-caspase-8(1:1000), anti-cleaved caspase-8(1:1000) and anti-GAPDH (1:2000) at 4°C overnight. After washing three times with TBST, the membranes were incubated with goat anti-rabbit secondary antibody (1:3000; Proteintech Group, Inc.) for 1 h at RT. The immune-active bands were detected by fluorography using ECL (enhanced chemiluminescence) reagents and quantified by Image Lab image acquisition and analysis software (Bio-Rad).

### Propidium Iodide staining and TUNEL staining

Mice received 10 mg/kg propidium iodide (PI; Sigma) through the jugular vein to label necrotic cells. After 2 h, the mice were sacrificed, and lung tissue was flushed with 5 ml of PBS via a needle into right ventricle three times. Then, the tissue was frozen at -80°C. Frozen sections of 10 um were cut and counterstained with DAPI for 2 min. Then, the necrotic cells were quantified under an Olympus IX71 microscope. Before TUNEL staining, the lungs were also flushed as mentioned above. TUNEL staining was conducted using a TUNEL detection kit according to the manufacturer’s instructions. Data analysis was performed using the Cell^P imaging software (Olympus, USA).

### Immunohistochemistry

After deparaffinization, lung sections were hydrated and epitope retrieval was performed according to the manufacturer’s protocol (DakoCytomation Target Retrieval Solution, pH 6.0; DakoCytoma- tion, Carpinteria, CA, USA). The lung sections were stained with rabbit polyclonal anti-mouse RIP3(Abcam ab56164), followed by secondary HRP-labeled polymer with DAB staining (EnVision + System-HRP, Dako), and hematoxylin counterstaining. Cellular morphology was identified by a pathologist who was initially blinded to study groups as reported[24–27].

### Collection of bronchoalveolar lavage fluid

Bronchoalveolar lavage fluid (BALF) was obtained by washing the airways of the right lungs three times with a total of 1.5 ml of PBS through a tracheal cannula. BALF was pooled and centrifuged at 4°C and 1500 r for 10 min. The supernatant was harvested for the neutrophil counts and the total protein concentration assay.

### ELISA and myeloperoxidase (MPO) measurement

TNF-a, IL-6, IL-1β, IL-1α, IL-10 ELISA kit and MPO assay kits were purchased from Nanjing Jiancheng Bioengineering Institute (Nanjing, China). The HMGB1 ELISA kit was obtained from R&D Systems. The BALF cytokine level and lung tissue MPO activity were measured according to the manufacturer’s instruction.

### Temperature monitoring and survival analysis

The ambient room temperature was maintained at 23°C. The mice were separated into individual containers 1 h prior to the measurements. The temperature was measured by inserting the probe 2 cm into the rectum via a lubricated digital rectal probe.

For survival study, The RIP3-WT and RIP3-KO mice were instilled with 30 mg/kg LPS. Health and behavior of the mice were assessed every 6 h up to 24 h. Upon presentation of defined criteria associated with lung injury progression (abnormal feeding behavior, diminished response to stimuli and failure to thrive), the mice were humanely euthanized according to approved IACUC guidelines (the use of cervical dislocation as a method of euthanasia). The survival time was recorded starting from 6 h after LPS instillation to 24 hours.

### Statistical analysis

Statistical analysis was performed using the GraphPad Prism 5.01 (GraphPad Software, San Diego, CA). The data were presented as mean±s.e.m. All of the data were analyzed by one-way ANOVA. Statistical analyses were performed using Student’s t-test for two groups and by ANOVA followed by Tukey’s post hoc test for multiple groups The survival curves were compared using the log-rank (Mantel-Cox) test. P<0.05 were considered statistically significantly.

## Results

### 1 The severity of lung injury in LPS-induced ARDS is concentration-dependent

**Fig 1:**
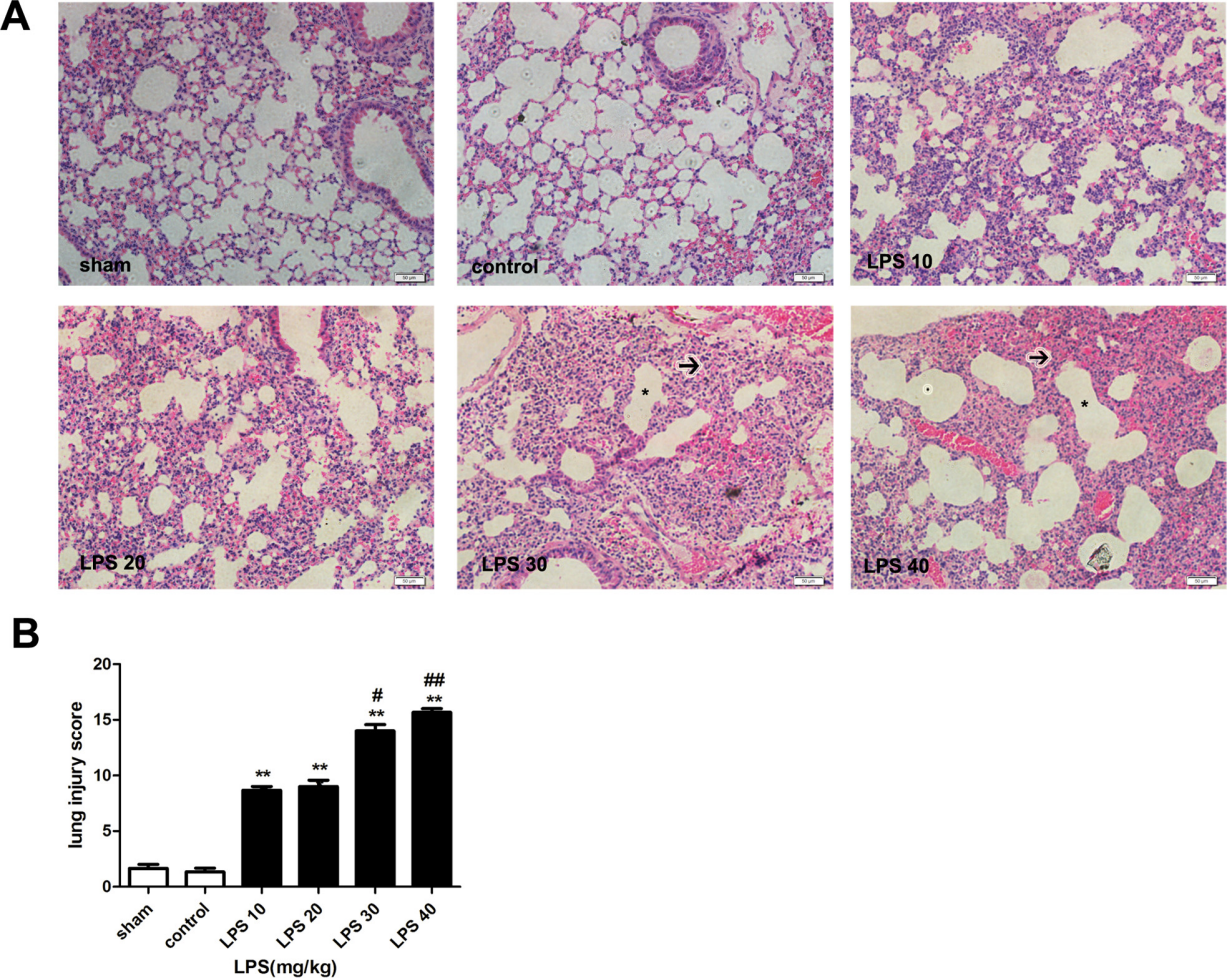
The severity of lung injury in LPS-induced ARDS is concentration-dependent. (A) H&E staining of lung tissues under different doses of LPS administration (200×). Atelectasis was marked with black arrow and alveolar fusion was marked with *. (B) Lung injury score under different doses of LPS administration. The mice were instilled with different concentrations of LPS at doses of 10 mg/kg~40 mg/kg, respectively, while the sham group was instilled with nothing, and the control group was instilled with PBS. The mice were sacrificed 24 h after LPS instillation, and the left lungs were obtained for H&E. The data were presented as mean±s.e.m; (n = 5). **P<0.01 versus the control group; ^#^P<0.05, ^##^P<0.01 versus the LPS 10 mg/kg group.

As shown in Fig 1, compared to sham or control groups, LPS, in all the four concentrations used, induced various degree’s lung injury in ARDS groups manifested as marked alveolar congestion, hemorrhage, elevation of infiltration of neutrophils in the airspace and vessel wall, and increase of thickness of the alveolar wall/hyaline membrane formation. Moreover, LPS-induced lung injury was concentration-dependent and peaked at the doses of 30 or 40 mg/kg evidenced as most severe pulmonary morphologic changes shown as larger area of atelectasis and alveolar fusion and the increased lung injury scores. Thus, treatment with 30 mg/kg LPS was chosen to induce severe ARDS in the ensuring experiments.

### 2 Apoptosis was increased in low dose LPS-induced mild ARDS but was decreased in high dose LPS-induced server ARDS

**Fig 2:**
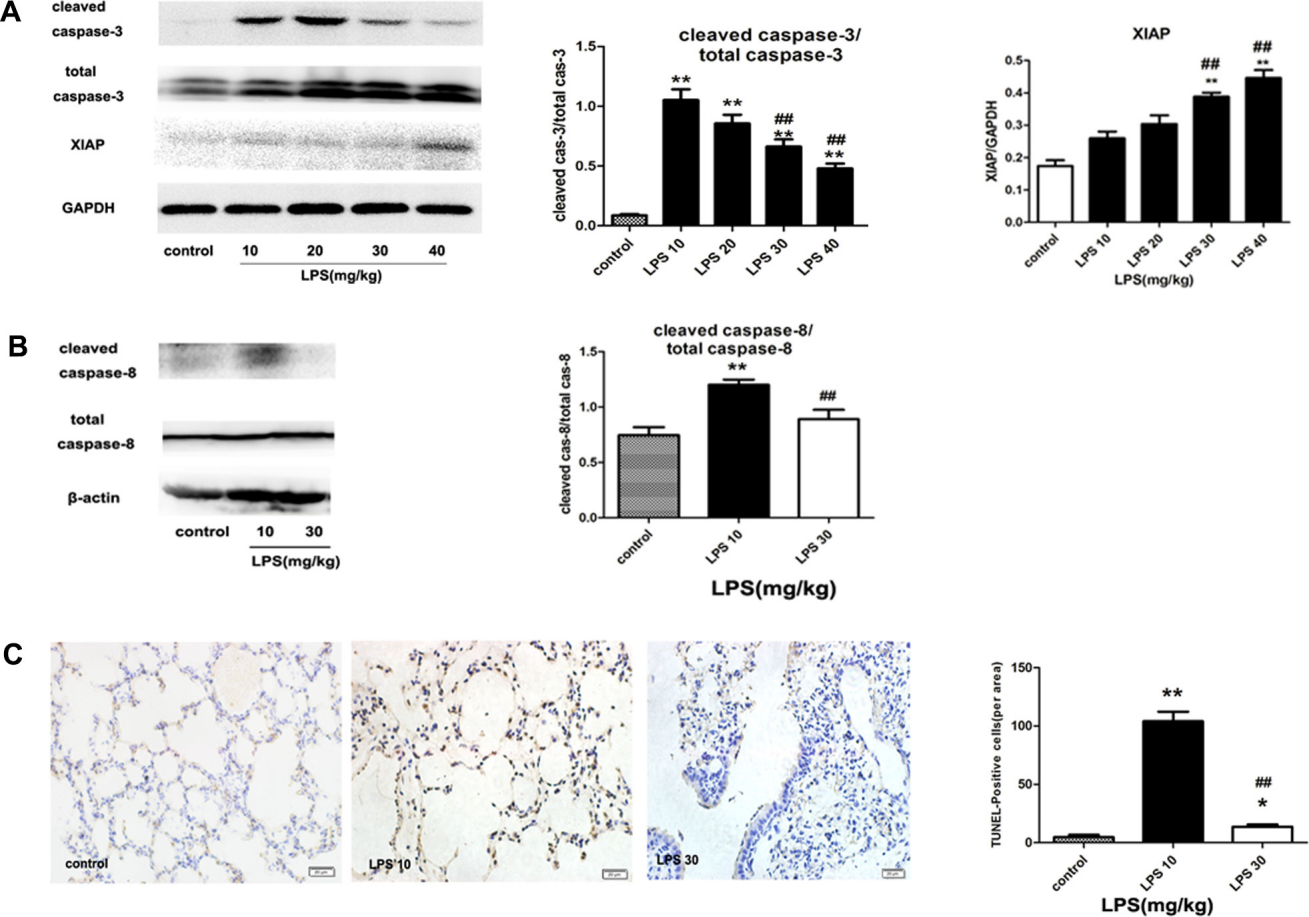
Apoptosis was increased in low dose LPS-induced mild ARDS but was decreased in high dose LPS-induced server ARDS. (A) The expression of total and cleaved caspase-3 and XIAP as detected by western blotting. (B) The expression of total and cleaved caspase-8 as detected by western blotting. (C) The number of apoptotic cells by TUNEL staining (400×). (D) TUNEL-positive cell counts per area. The mice were instilled with different concentrations of LPS at doses of 10 mg/kg~40 mg/kg, respectively, while the control group was instilled with PBS. The mice were sacrificed 24 h after LPS instillation. The data are presented as mean±s.e.m; (n = 5). *P<0.05, **P<0.01 versus the control group; ##P<0.01 versus the LPS 10 mg/kg group.

Apoptosis has been considered as the prevalent type of cell death in acute lung injury[28, 29]. The changes of apoptosis in the lung were determined in our LPS-induced ARDS. As shown in Fig 2A, cleave caspase-3, marker of apoptosis, was significantly increased in low dose (10 mg/kg and 20 mg/kg) LPS treatment groups but was decreased when LPS dose increased to 30 mg/kg and returned to a level comparable to the control group when LPS dose increased to 40 mg/kg. Consistently, cleaved caspase-8 expression was significantly enhanced in low dose LPS treatment group but was reduced when the dosage of LPS increased to relative higher doses (Fig 2B). These results were confirmed by TUNEL staining, which showed that number of TUNEL-positive cells was increased in 10 mg/kg LPS treatment groups but was decreased when LPS concentration increased to 30 mg/kg (Fig 2C).

XIAP can inhibit apoptosis[11]. As shown in Fig 2A, XIAP expression was significantly increased in LPS 30 and 40 mg/kg groups compared to that of the control group and to that in the LPS 10 mg/kg group.

### 3 Lung tissue necroptosis was increased in high dose but not in low dose LPS-induced ARDS

**Fig 3:**
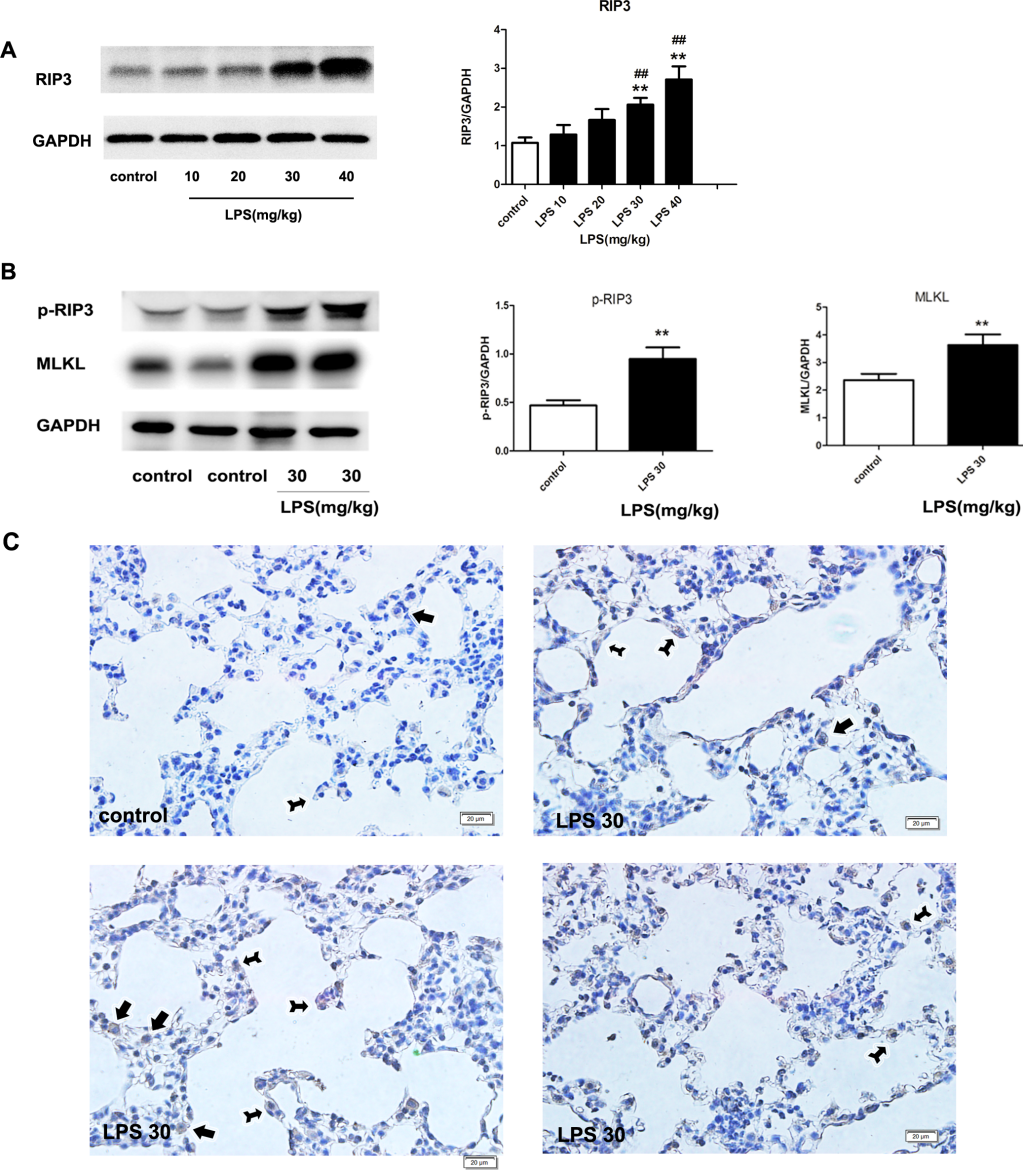
Necroptosis was increased in the lung in high dose but not in low dose LPS-induced ARDS. (A) The expression of RIP3 as detected by western blotting. (B) p-RIP3 and MLKL expression as detected by western blotting. (C) Immunohistochemistry of lung section for detecting of RIP3. RIP3 was increased in the cytoplasm of alveolar wall epithelial cells (as directed by black arrow with bifurcated tail) and macrophage-like cells (as directed by black arrow without bifurcated tail). The mice were instilled with different concentrations of LPS at doses of 10 mg/kg~40 mg/kg, respectively, while the control group was instilled with PBS. The mice were sacrificed 24 h after LPS instillation. The data are presented as mean±s.e.m; (n = 5). **P<0.01 versus the control group; ##P<0.01 versus the LPS 10 mg/kg group.

As shown in Fig 3A, we found that the expression of RIP3, marker of necroptosis, was significantly increased in high dose LPS (30 mg/kg and 40 mg/kg) but not in low dose LPS (10 mg/kg and 20 mg/kg) groups. In the process of necroptosis, RIP1 and RIP3 phosphorylate each other, and MLKL functions as a substrate in RIP3-mediated necroptosis. As shown in Fig 3B, both phosphorylated RIP3 (p-RIP3) and MLKL expression increased significantly 24 h after 30 mg/kg LPS administration compared to that of the control group.

The RIP3-immunohistochemical staining was applied to identify the cellular sources of RIP3 in the airway in our severe ARDS model (LPS 30 mg/kg). Cellular morphology was identified by a pathologist who was initially blinded to study groups as reported[24–27]. As shown in Fig 3C, RIP3 was increased in the cytoplasm of alveolar wall epithelial cells and macrophage-like cells.

### 4 RIP3 depletion ameliorated lung injury and improved survival rate in LPS-induced ARDS in mice

**Fig 4:**
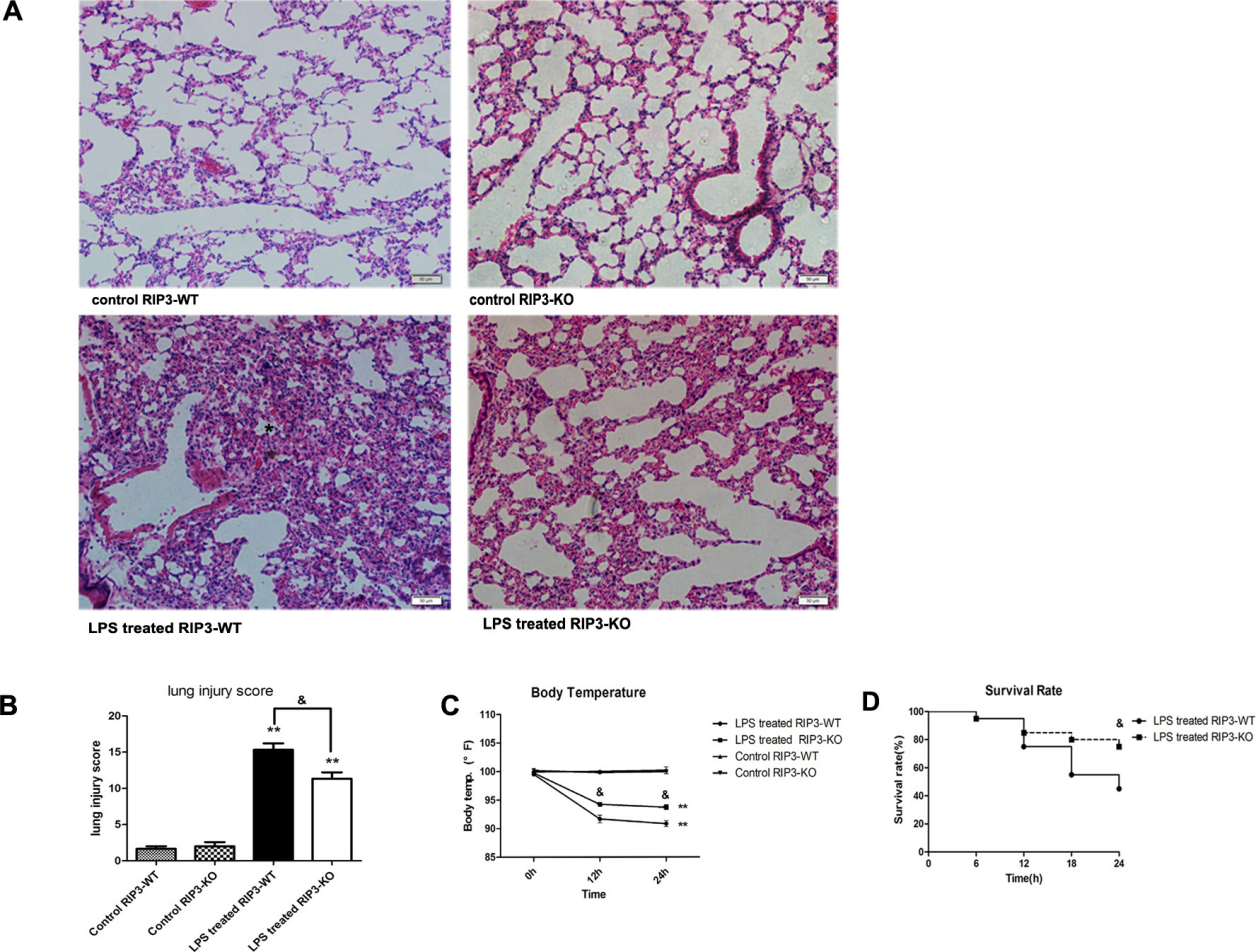
RIP3 depletion ameliorated lung injury and improved survival rate in LPS-induced ARDS in mice. (A-B) Pathological changes of lung tissues as determined by H&E staining (200×, n = 5). (C) Body temperature of LPS treated RIP3-WT and RIP3-KO mice (n = 10~16). (D) Survival rate of LPS treated RIP3-WT and RIP3-KO mice (n = 20). RIP3-WT and RIP3-KO mice were both divided into control and LPS groups, respectively. The LPS group mice were instilled with 30 mg/kg LPS, while the control group mice were instilled with PBS. The mice were sacrificed 24 h after LPS instillation. The data are presented as mean±s.e.m; **P<0.01 versus the respective control group; &P<0.05 versus the LPS treated RIP3-WT group.

We have demonstrated that in high dose LPS-induced severe ARDS, apoptosis was decreased while necroptosis was increased, suggesting that necroptosis played a critical role in LPS-induced severe ARDS. High dose LPS was applied in RIP3-KO mice to induce severe ARDS in order to confirm the role of RIP3 in lung injury in severe ARDS model and to explore the underlying mechanism. As shown in Fig 4A and 4B, compared to that in WT control mice, lung injury was attenuated in high dose LPS treated RIP3-KO mice evidenced as minor morphological changes that was associated with reduced lung injury score and decreased inflammatory cell infiltration and less severer alveolar wall collapse. LPS can induce hypothermia symptoms in ARDS. As shown in Fig 4C, the body temperature in LPS treated RIP3-KO mice was significantly higher than that in LPS treated RIP3-WT mice at both 12 h and 24 h. In addition, as shown in Fig 4D, the survival rate of LPS-treated RIP3-KO mice was higher than that in LPS treated RIP3-WT mice.

### 5 RIP3 depletion decreased the levels of IL-1α, IL-1β, IL-6 and HMGB1 in BALF in LPS-induced ARDS

**Fig 5:**
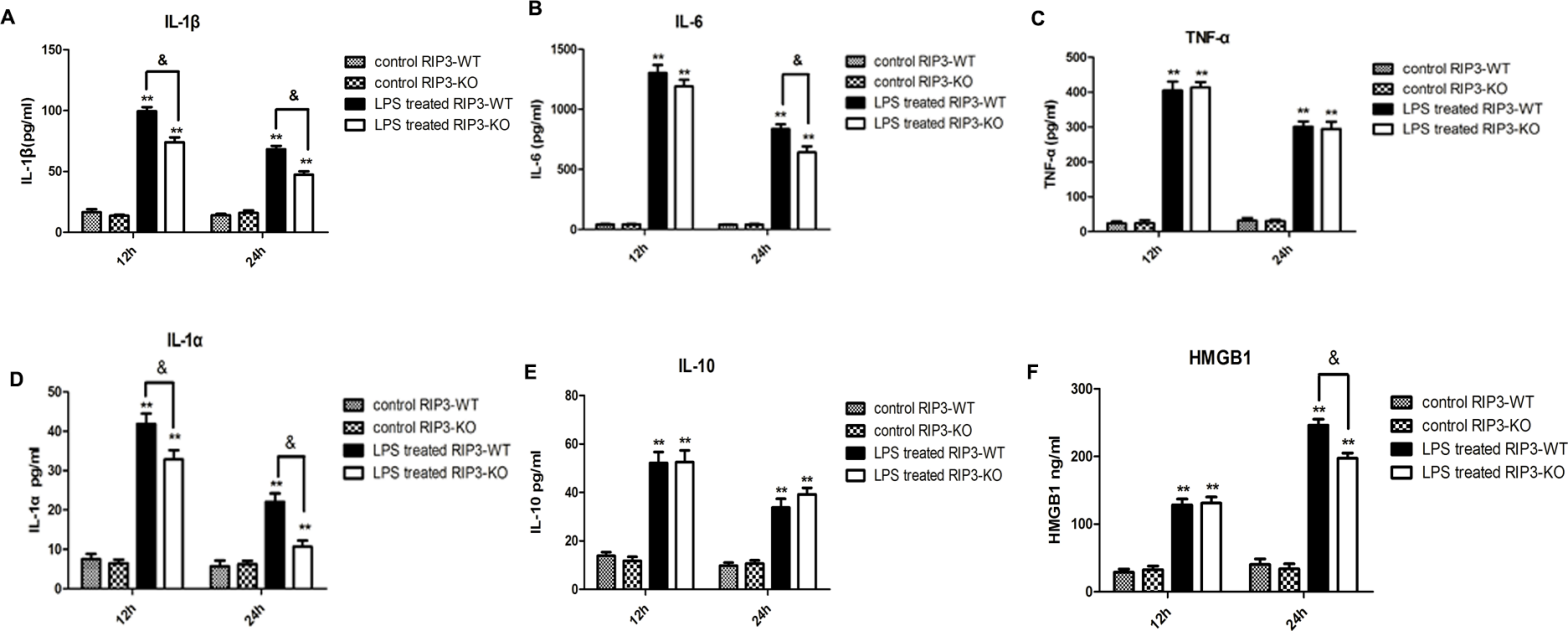
RIP3 depletion decreased the levels of IL-1α, IL-1β, IL-6 and HMGB1 in BALF in LPS-induced ARDS. (A) The concentration of IL-1β in BALF. (B) The concentration of IL-6 in BALF. (C) The concentration of TNF-α in BALF. (D) The concentration of IL-1α in BALF. (E) The concentration of IL-10 in BALF. (F) The concentration of HMGB1 in BALF. RIP3-WT and RIP3-KO mice were divided into two groups. The LPS group mice were instilled with 30 mg/kg LPS, while the control group mice were instilled with PBS. The mice were sacrificed at 12 h or 24 h after LPS instillation. The data are presented as mean±s.e.m; (n = 10). **P<0.01 versus the respective control group; ^&^P<0.05 versus the LPS treated RIP3-WT group.

In ARDS, pro-inflammatory cytokines mediate the inflammatory response to LPS and promote damage to the lung[30]. Necrosis is associated with a high level of pro-inflammatory cytokines[31] and intracellular contents such as DAMPs release[5]. HMGB1 is a kind of DAMP that can be released to the extracellular matrix passively in necroptosis occurrence[32]. Therefore, the classical pro- and anti-inflammatory cytokines IL-1α, IL-1β, IL-6, TNF-α, IL-10 and HMGB1 levels were measured in BALF. As shown in Fig 5A–5F, LPS treatment significantly increased IL-1α, IL-1β, IL-6, TNF-α, IL-10 and HMGB1 levels in both LPS treated RIP3-WT and LPS treated RIP3-KO groups. In addition, when compared with LPS treated RIP3-WT mice, the levels of IL-1α and IL-1β in LPS treated RIP3-KO mice decreased at both 12 h and 24 h, IL-6 and HMGB1 also decreased at 24 h, but TNF-α and IL-10 did not decrease significantly in the LPS treated RIP3-KO group.

### 6 RIP3 depletion reduced the tissue MPO activity, neutrophil counts and total protein concentration in BALF in LPS-induced ARDS

**Fig 6:**
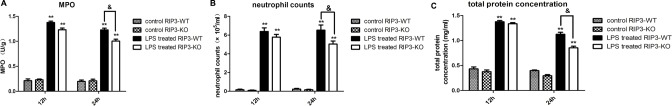
RIP3 depletion reduced the tissue MPO activity, neutrophil influx and total protein concentration in BALF in LPS-induced ARDS. (A) The MPO activity in lung tissue. (B) The neutrophil counts in BALF. (C) The total protein concentration in BALF. RIP3-WT and RIP3-KO mice were divided into two groups. The LPS group mice were instilled with 30 mg/kg LPS, while the control group mice were instilled with PBS. The mice were sacrificed 12 h or 24 h after LPS instillation. The data were presented as mean±s.e.m. (n = 10). **P<0.01 versus the respective control group; ^&^P<0.05 versus the LPS treated RIP3-WT group.

The recruitment of circulating neutrophil into the lung greatly contributes to the development and progression of ARDS[33, 34], while release of DAMP such as HMGB1, IL-1α/β, IL-6 promote this process forcefully[35]. In our study, the influx of neutrophil was studied at 12 h and 24 h after LPS administration. As shown in Fig 6A and 6B, the LPS treated RIP3-KO mice showed a reduction in tissue MPO activity and BALF neutrophil counts at 24 h compared with LPS treated RIP3-WT mice. Neutrophil recruitment could increase the pulmonary protein leakage by releasing proteases to punch the endothelial-epithelial barrier thereby increased the microvascular permeability in the lung[33, 34]. As shown in Fig 6C, the LPS-treated-RIP3-KO mice showed a reduction of BALF total protein concentration at 24 h compared to that of the LPS treated RIP3-WT mice.

### 7 RIP3 depletion reduced the necrotic cells in the lung and decreased the expression of MLKL, but had no impact on cleaved caspase-3 in LPS-induced ARDS

**Fig 7:**
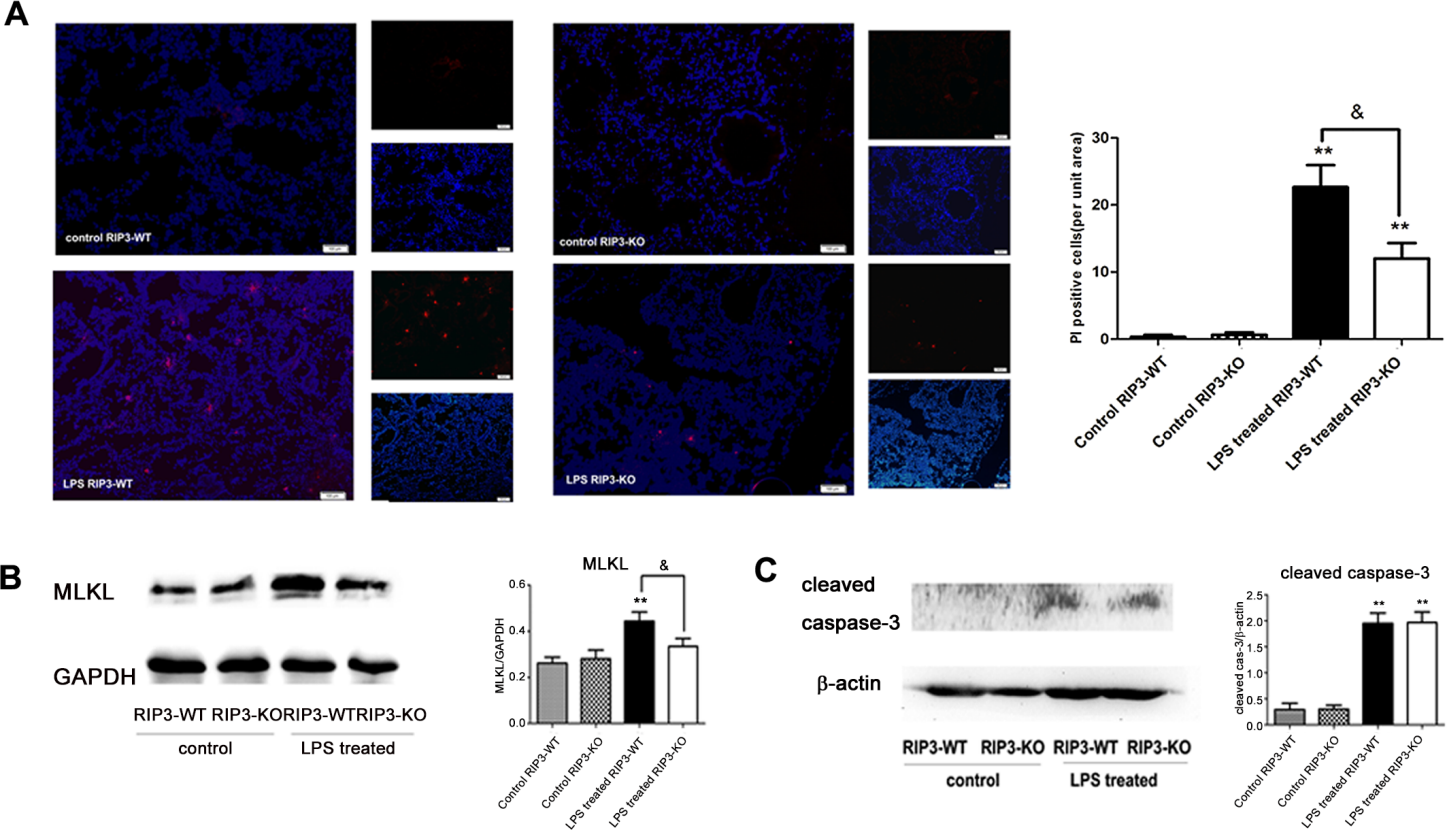
RIP3 depletion reduced the necrotic cells in the lung and decreased the expression of MLKL, but had no impact on cleaved caspase-3 in LPS-induced ARDS. (A) The number of necrotic cells as determined by PI staining (100×). Frozen sections were stained with propidium iodide (PI, red) and nuclear stain (DAPI, blue). The cells were counted by the Cell^P imaging software. (B) MLKL expression as detected by western blotting. (C) Cleaved caspase-3 expression as detected by western blotting. RIP3-WT and RIP3-KO mice were divided into control and LPS groups, respectively. The LPS group mice were instilled with 30 mg/kg LPS, while the control group mice were instilled with PBS. The mice were sacrificed 24 h after LPS instillation. The data are presented as mean±s.e.m; (n = 10). **P<0.01 versus the respective control group; ^&^P<0.05 versus the LPS treated RIP3-WT group.

Necroptosis is a kind of programmed necrosis which can be detected by nuclear PI-positive staining. As shown in Fig 7A and 7B, the number of necrotic cells in both LPS treated RIP3-WT and LPS treated RIP3-KO mice increased compared with their respective controls (control RIP3-WT and control RIP3-KO), but the LPS treated RIP3-KO group showed a reduction of necrotic cells compared to those in LPS treated RIP3-WT group.

As shown in Fig 7B, the MLKL expression in the LPS treated RIP3-WT group was significantly increased compared to that of the control RIP3-WT group but decreased to normal level in the LPS treated RIP3-KO group compared to that of the LPS treated RIP3-WT and control RIP3-KO groups. As shown in Fig 7C, LPS induced the expression of cleaved caspase-3 in both LPS treated RIP3-WT and LPS treated RIP3-KO group when compared with the control groups. However, the expression of cleaved caspase-3 did not significantly differ between the LPS treated RIP3-WT and LPS treated RIP3-KO group.

## Discussion

We have shown in the current study that in high dose LPS-induced severe ARDS, lung injury was mainly due to RIP3-mediated necroptosis, while in low dose LPS-induced mild ARDS, lung injury was mainly a consequence of caspase-dependent apoptosis. We have provided evidences that RIP3 depletion can reduce LPS-induced inflammation response (reduced pro-inflammatory cytokines release, decreased tissue MPO activity, decreased neutrophil influx and total protein concentration in BALF), attenuate LPS-induced lung injury, and alleviate hypothermia symptoms, which eventually improve survival rate in high dose LPS-induced severe ARDS in RIP3-KO mice. We further showed that these beneficial effects of RIP3 depletion may be possibly through reducing MLKL activation and the subsequently reduce necroptosis with concomitant reductions in pulmonary inflammation mice with LPS-induced severe ARDS.

Necrosis occurs when cells are exposed to a high concentration of detergents, oxidants, ionophores, or severe pathological insults[3]. Necroptosis, a programmed cell death, by inducing cell necrosis plays important roles in severe tissue injury in many diseases including myocardial ischemia reperfusion injury[36] and lung injury[19]. However, the mechanism whereby necroptosis induces lung injury in ARDS has not been explored. Studies have shown that the execution of necroptosis needs RIP3 phosphorylation, and recent studies verified that MLKL was a downstream protein of RIP3. In our study, we observed that p-RIP3 and MLKL were significantly increased after LPS administration that was associated with increased cell necrosis. RIP3 was increased in the cytoplasm of alveolar epithelial cells and macrophage-like cells in mice with severe ARDS. Studies by Gandhirajan and co-workers have shown that RIP3 expression was increased in pulmonary endothelium in an LPS-induced systemic vascular inflammation mouse model[18]. Although have not observed the expression of RIP3 in pulmonary endothelium in the current study, the findings of the current study do provide new insights into the cellular mechanism of RIP3-mediated lung injury in severe ARDS. Further, RIP3-KO mice showed decreased MLKL and decreased cell necrosis after high dose LPS administration, a situation when apoptosis was returned to normal as compared to control group. Results from our study that apoptosis was inhibited and RIP3 and MLKL (markers of necroptosis) were increased in severe ARDS induced by the administration of high dose LPS strongly suggests that the increase of necrosis in our high dose LPS-induced lung injury was mainly due to the induction of necroptosis. However, how MLKL mediated LPS-induced necroptosis remains unclear. Studies have shown that MLKL forms a homotrimer through its amino-terminal coiled-coil domain and plays cortical roles in TNF-induced necroptosis[37]. Therefore, the mechanism whereby MLKL mediates necroptosis following its activation/stimulation by PIP3 to cause/exacerbate lung injury under the circumstance of high dose LPS exposure needs to be further studied.

A recent study from Kipyegon Kitur and coworker reported that Staphylococcus aureus USA300 strains cause a highly inflammatory necrotizing pneumonia[16], and knockout of RIP3 significantly improved staphylococcal clearance and retained an alveolar macrophage population and decreased release of pro-inflammatory cytokines such as KC, IL-6, TNF, IL-1α and IL-1β[16]. In TNF-α induced mouse systematic sepsis model, RIP3-KO mice recovered from hypothermia and survived longer, which was partly by reducing DAMPs and IL-1β, IL-6[14]. In our current study, we found that in high dose LPS (30 mg/kg) induced severe ARDS, depletion of RIP3 by reducing MLKL decreased necroptosis and subsequently ameliorated the lung injury, increased body temperature, improved survival rate, and decreased the expression of IL-1α/β and IL-6. These together with the fact that RIP3 is essential for the induction of necroptosis indicate that RIP3-dependent necroptosis promotes LPS induced lung injury in severe ARDS through enhancing inflammation. Of note, depletion of RIP3 had no impact on the secretion of TNF-a and IL-10 in BALF in LPS-induced ARDS. Studies have shown that in TNF-α induced mouse systematic sepsis model, RIP3 is not directly involved in pro-inflammatory cytokines transcriptions, but could sustain their secretion indirectly by inducing cell necrosis which releases DAMP[14]. In ARDS model, NF-*k*B plays the dominant role in encoding the transcriptional activation of pro-inflammatory cytokines and chemokines[38], and RIP3 is dispensable for normal NF-*k*B[39]. These suggest that in order to sustain the release of pro-inflammatory cytokines (such as TNF-α) in ARDS, both normal function of NF-*k*B and necrosis-mediated sustain DAMP release all required. This may why in our current study, not all pro-inflammatory cytokines decrease at every time point in the RIP3-KO mice, although we observed that IL-1α and IL-1β decreased obviously at all time points in our ARDS model. Of note, studies by Vince JE et al have shown that RIP3 is a regulator of IL-1 activation[12]. This finding may suggest that the protective effects of RIP3 depletion in severe ARDS mice seen in our study might be achieved, at least in part, through reducing IL-1 activation.

Many studies support that necroptosis can release a variety of intracellular contents, such as HMGB1, LDH and HSP, which can promote neutrophil infiltration[40, 41]. HMGB1 can strongly promote neutrophil migration in ARDS[42, 43]. In our study, RIP3 depletion reduced the necrotic cell counts in lung tissue and the HMGB1 release into BALF. The neutrophil cell counts in BALF and MPO activity in lung tissue were also reduced at 24h in RIP3-KO mice. Studies have demonstrated that the invading neutrophils play a very important role in ARDS process, as capable of releasing proteases to cause tissue damage and interrupting endothelial-epithelial cell junctions to increase microvascular permeability in the lung. The increased permeability of alveolar walls could cause protein leakage to alveolar space[33, 34]. Therefore, we further investigated the effects of RIP3 depletion on alveolar protein leakage. Our results showed that both neutrophil infiltration into lung tissue and protein leakage into BALF were decreased at 24 h in LPS treated RIP3-KO mice. These results suggested that RIP3-mediated necroptosis promotes high dose LPS-induced inflammation through enhancing the lung neutrophil infiltration and protein leakage in mice.

The most novel finding of our current study is that in high dose LPS-induced severe ARDS, lung injury was mainly due to RIP3-mediated necroptosis, while in low dose LPS-induced mild ARDS, lung injury was mainly due to caspase-dependent apoptosis. In the present study, we showed that in LPS-induced mild ARDS, apoptosis was increased with no change of necroptosis, while in LPS-induced severe ARDS, apoptosis was inhibited and necroptosis was increased, providing a clue that negative regulation may existed between apoptosis and necroptosis in LPS-induced ARDS. Studies have shown that the cleavage of caspase-3/8 was involved in apoptosis, while total caspase-8 cleaved RIP1-RIP3 complex and subsequently suppressed necroptosis[5]. In our study, we observed that caspase 3/8-dependent apoptosis was increased significantly in low dose LPS-induced mild ARDS, while it was inhibited massively under high dose LPS-induced severe ARDS. Moreover, studies also showed that necroptosis serves as an alternative type of cell death when caspase-dependent apoptosis is inhibited or absent, and that apoptosis can inhibit RIP3-mediated necroptosis[5]. Therefore, our results suggested that apoptosis may play a dominant role in mild ARDS, while in severe ARDS, apoptosis is inhibited, thereby increases necroptosis and the subsequent induction of inflammation and lung injury in ARDS. These were further confirmed in our study, in which we showed that the prototype member of IAP family XIAP, which inhibits apoptosis by suppressing caspase-3 and caspase-7[11], was progressively increased when dose of LPS increased in our ARDS model. This together with the finding that apoptosis was inhibited in severe ARDS, suggests that the increase of XIAP might lead to the inhibition of apoptosis in severe ARDS and the subsequent induction of necroptosis. However, the potential interplay between apoptosis and necroptosis and the underlying mechanism in different dosage of LPS-induced ARDS merits further study.

In conclusion, our results indicate that RIP3-mediated necroptosis through enhancing inflammation induces lung injury in high dose LPS induced severe ARDS. Inhibiting RIP3 could protect mice against LPS-induced ARDS, at least in part, through reducing lung necrosis, decreasing inflammation and HMGB1 release in BALF, and weakening the lung neutrophil infiltration and protein leakage. Results from the present study provide insight into the effects and mechanisms of RIP3 inhibition in the treatment of ARDS, which may lead to the development of effective therapeutic regimens in combating LPS-induced ARDS.

## Abbreviations

ARDS: acute respiratory distress syndrome
RIP3: receptor interacting protein 3
MLKL: mixed lineage kinase domain-like protein
PI: propidium Iodide
HMGB1: high-mobility group box 1
XIAP: X-linked inhibitor of apoptosis
DAMPs: damage-associated molecular pattern
